# Consistency of information on the provision of water fluoridation in Brazil

**DOI:** 10.1590/1980-549720240029

**Published:** 2024-06-14

**Authors:** Anna Laura Santos Doalto, Lorrayne Belotti, Camila de Moraes Paulino, Paulo Frazão

**Affiliations:** I Universidade de São Paulo, School of Public Health, Department of Policy, Management and Health – São Paulo (SP), Brazil.; II Hospital Israelita Albert Einstein, Center for Studies, Research and Practice in Primary Care and Networks – São Paulo (SP), Brazil.

**Keywords:** Public policy, Water fluoridation, Health surveillance, Water supply

## Abstract

**Objective:**

This study aimed to assess the consistency of data regarding the provision of fluoridation in Brazilian municipalities with water supply systems.

**Methods:**

Official data from the National Basic Sanitation Survey and the National Information System on Sanitation for 2017 were compared.

**Results:**

Out of 5,570 municipalities in Brazil, 4,546 (81.6%) had water supply systems. The agreement between data sources was 84%, with a Kappa of 0.668, indicating substantial agreement. However, the estimates of fluoridation provision exhibited an average discrepancy of 8.1 percentage points, ranging from 1.2 points in the Central-West region to 21.4 points in the Northeast region.

**Conclusion:**

To address these inconsistencies, it is essential to enhance information sources, ensuring more reliable data for health, sanitation authorities, and society at large.

## INTRODUCTION

A thorough comprehension of population health and the evaluation of intersectoral health policies’ impact fundamentally rely on consistent data regarding health determinants. These determinants serve as the groundwork for formulating and executing public policies that affect the health status of communities, encompassing socioeconomic, environmental, behavioral factors, and access to health services^1^.

One of these determinants encompasses the provision of treated and fluoridated water, which is a public health strategy known to effectively prevent and reduce tooth decay. However, its efficacy is intricately tied to monitoring the quality of drinking water concerning the fluoride parameter and relies on accurate information regarding its supply. Ensuring the effectiveness of its provision is a pivotal step in promoting oral health on a population level and mitigating inequalities in access to dental care^2^. This is particularly critical in Brazil, where public spending on oral health has been limited to 0.07% of gross domestic product (GDP)^3^, whereas in some European countries with similar economies, it has reached 0.12% of GDP^4^.

The objective was to assess the consistency of information regarding fluoridation coverage in Brazilian municipalities with water supply systems in 2017, based on two official sources of information.

## METHODS

Descriptive study utilizing openly available official data from the year 2017 sourced from the following:

1. National Basic Sanitation Survey (*Pesquisa Nacional de Saneamento Básico –*PNSB)^5^: An initiative led by the Brazilian Institute of Geography and Statistics (*Instituto Brasileiro de Geografia e Estatística* – IBGE) aimed at gathering data from entities responsible for water supply and sewage services across Brazilian municipalities. This survey encompasses registration and operational details concerning water capture, collection, treatment, distribution, and billing. PNSB facilitates assessments of service provision and quality, as well as analysis of environmental conditions, directly impacting population health and quality of life.
2. National Sanitation Information System (*Sistema Nacional de Informações sobre Saneamento* – SNIS)^6^: A database administered by the Sanitation Secretariat, under the Ministry of Regional Development in 2017. It is designed to compile institutional, administrative, operational, managerial, economic-financial, accounting, and quality-related information regarding the provision of water, sewage, and urban solid waste management services. The platform serves as a reference for comparing and assessing service performance. Data is collected annually from service providers or municipal bodies responsible for managing these services. The database is publicly accessible free of charge through a dedicated electronic platform.

Initially, the study encompassed all 5,570 Brazilian municipalities. Subsequently, municipalities with water supply systems (WSS) were categorized based on the provision of fluoridation. They were classified into three distinct categories: “yes,” indicating the provision of fluoridation; “no,” indicating its absence; and “no information,” for cases where data was unavailable.

Data analysis entailed processing records using the municipal code and summarizing the proportion of municipalities ensuring the provision of public policy according to each data source. The difference in coverage of the strategy provision (in percentage points) was then calculated for the country and each geographic region. To assess the agreement between the data sources from PNSB and SNIS, a percentage agreement was utilized, ranging from 0 to 100%, and the Kappa statistic, varying from 0.0 to 1.0, alongside corresponding intervals of 95% confidence. The values were interpreted based on the following criteria: 0.01 to 0.20 for slight agreement, 0.21 to 0.40 for fair agreement, 0.41 to 0.60 for moderate agreement, 0.61 to 0.80 for substantial agreement, and 0.81 to 1.0 for almost perfect agreement.

## RESULTS

**Figure 1:**
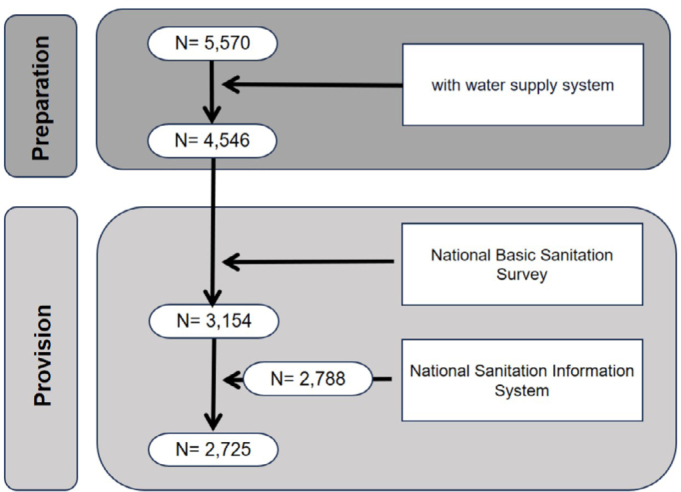
Data flow in information sources.

Of the 5,570 Brazilian municipalities, 4,546 had WSS. In Figure 1, the data flow illustrates the cross-referencing of information from both sources. According to data from the PNSB, water fluoridation was provided in 69.4% (3,154) of municipalities with WSS, while, according to SNIS, 61.3% (2,788) received the benefit, reflecting a discrepancy of 8.1 percentage points (Table 1), with significant variation between Brazilian geographic regions (1.2 pp. for the Central-West to 21.4 pp. for the Northeast). The percentage agreement was 84.1%, with lower values in the North (78.7%) and Northeast (67.1%) regions. The Kappa statistic was 0.668 (95% CI: 0.648-0.689), indicating substantial agreement. This value was lower than 0.61 in the North, Northeast, and South regions.

**Table 1:** Coverage of provision for fluoridation in municipalities with water supply systems according to official sources of information in 2017 and geographic regions. Difference in percentage points and agreement values.

Municipalities with WSS	Fluoridation coverage			Dif. pp.	PC	Kappa	LL	UL	Meaning
	PNSB		SNIS						
Brasil	4,546	69.4	61.3	8.1	84.1	0.668	0.648	0.689	Substantial
North	287	18.8	17.4	1.4	78.7	0.540	0.442	0.638	Moderate
Northeast	1,168	37.9	16.5	21.4	67.1	0.324	0.278	0.371	Regular
Southeast	1,616	88.3	84.8	3.5	92.6	0.692	0.646	0.738	Substantial
South	1,049	94.6	89.7	4.9	90.2	0.338	0.252	0.424	Regular
Central-West	426	55.9	54.7	1.2	86.6	0.751	0.696	0.806	Substantial

WSS: Water Supply Systems; PNSB: Pesquisa Nacional de Saneamento Básico, 2017; SNIS: Sistema Nacional de Informação de Saneamento, 2017; Dif. pp.: Percentage Point Difference; PC: Percentage Concordance; LL: Lower Limit; UL: Upper Limit.

## DISCUSSION

The consistency of information regarding the provision of water fluoridation in supply systems was substantial nationwide. However, lower percentage agreement was observed in the North and Northeast regions. Additionally, the difference in strategy coverage between information sources exceeded 20 percentage points in the Northeast region. Both sources relied on declarations from water supply service providers, making it impractical to adopt one as a reference over the other. The findings underscore the necessity of supplementing provider declarations with additional data and highlight significant opportunities for enhancing information regarding the provision of this strategy and intersectoral management of public policy.

The percent agreement metric indicates total agreement irrespective of chance, with the North and Northeast regions displaying lower values compared to the national average. Conversely, the Kappa statistic gauges agreement beyond chance, influenced by the asymmetry in agreement and disagreement between information sources. Therefore, the value obtained for the South region should not be a cause for concern given that 922 municipalities of the 1,049 with WSS belonging to the region were provided by public policy, according to both sources of information. Likewise, the higher Kappa statistic values observed for the Central-West region warrant scrutiny, considering that 225 out of 426 municipalities with WSS in the region received the benefit according to the compared sources. While the symmetry of values in this region tends to align the Kappa statistic closer to the percent agreement value, asymmetry may lead to discrepancies^7^.

Studies assessing information consistency benefit from a diverse array of available data. However, a significant limitation arises from the absence of direct water sample collection data from the distribution networks of WSS in the North, Northeast, and Central-West regions. Respectively, only 1.0, 13.9, and 9.6% of municipalities in these regions had health surveillance records on fluoride concentration in WSS^8^. The availability of such records would facilitate comparison with the self-declared data provided by both information sources.

The study concludes that while there is substantial consistency of information on water supply system fluoridation at the national level, there is an 8.1 percentage point discrepancy at the municipal level, with inconsistencies exceeding 20 percentage points in the Northeast region. These findings underscore the imperative to enhance information regarding public policy strategy and management. The seemingly straightforward measurement process poses a significant challenge for sanitation authorities, particularly in the Northeast region, where water resources are limited, and control procedures for water supply systems must maintain rigorous precision akin to other regions.
